# Precision and accuracy of craniofacial growth and orthodontic treatment evaluation by digital image correlation: a prospective cohort study

**DOI:** 10.3389/froh.2024.1419481

**Published:** 2024-07-26

**Authors:** Jan Christian Danz, Simone Stöckli, Christian Per Rank

**Affiliations:** ^1^ Department of Orthodontics and Dentofacial Orthopedics, School of Dental Medicine ZMK, University of Bern, Bern, Switzerland; ^2^ Orthodontics and Dentofacial Orthopedics, Odense, Denmark

**Keywords:** growth and development, structural superimposition, digital image correlation, orthodontic treatment, maxillofacial surgery

## Abstract

**Introduction:**

A precise and accurate method for structural superimposition is essential for analyzing dentofacial growth and orthodontic or surgical treatment in longitudinal studies. The errors associated with different superimposition methods have not yet been assessed in high-quality studies.

**Objectives:**

This study aimed to assess the precision and accuracy of digital image correlation (DIC) for structural superimposition.

**Methods:**

Two cephalometric images from 30 consecutive patients were superimposed using three DIC methods, each measured twice by two examiners. Areas including the contours of the sella, the whole cranial base (CB), and Walker's point and lamina cribrosa (WPLC) were compared using a random coefficient model. Inter-rater and intra-rater errors were assessed for each method.

**Results:**

WPLC provided the best precision for image rotation and cephalometric landmarks. Systematic bias was observed between the WPLC and CB methods for image rotation and most landmarks. The intra-rater error in image rotation during DIC was strongly correlated with the intra-rater error in the landmarks of the anterior nasal spine, articulare, and pogonion.

**Conclusion:**

Structural superimposition using DIC with WPLC is a precise method for analyzing dentofacial growth and orthodontic or surgical treatment. Moreover, the best method is the measurement of longitudinal dental and craniofacial changes on structurally superimposed cephalometric radiographs with WPLC and a reference grid including the true vertical and horizontal lines from Walker's point.

## Introduction

Superimposition of lateral cephalometric head films has been used in longitudinal studies on dentofacial growth and orthodontic or surgical treatment. A commonly used method is the superimposition of cephalometric landmarks, such as the sella and nasion, sella and gnathion, and nasion and basion (1). Superimpositions on cephalometric landmarks are easily computable and have been widely used by clinicians in computer-aided measurements (2–5). However, the use of these landmarks creates systematic error, especially in growing individuals, as they move in relation to the stable structures of the anterior cranial base (6). The anterior wall of the sella turcica, the lower contour of the anterior clinoid processes, cribriform plate of the ethmoid bone and frontoethmoidal crest, cerebral surfaces of the orbital roof, and details of the trabecular system in the ethmoidal bone become stable very early in life (7 years) (7–13). This early stability has been confirmed with a 95% likelihood of sphenoethmoidal suture closure at the age of 2.9 years in girls and at 8.1 years in boys (14).

Björk (8) developed a method to superimpose lateral cephalometric headfilms on templates of stable anatomical structures of the anterior cranial base (“The Structural Method”). Superimposition using hand-traced templates is time-consuming, subject to human error (15, 16), and dependent on personal experience, knowledge, and understanding of craniofacial anatomy (8, 17–20); additionally, the precision of current commercial software remains unknown (21, 22).

Instead of using landmarks or contours, digital image correlation (DIC) divides an image into sub-images (facets). These facets are then matched to a reference to track displacement and rotation (23, 24). DIC has been widely applied in biomechanics and has been used to analyze dental materials (25). Software developers (e.g., OnyxCeph3TM) have introduced DIC for cephalometric superimposition. However, studies investigating the precision or accuracy of cephalometric superimposition using DIC are lacking, and the errors of different superposition methods have not yet been assessed in high-quality studies (26).

This study aimed to test the precision and accuracy of DIC in assessing longitudinal growth and treatment changes in three different areas of the anterior cranial base. The secondary aims were to analyze errors during superimposition and to determine the degree to which the method, observer, reproducibility, and image rotation affected cephalometric landmarks.

## Materials and methods

### Participants and study design

Thirty consecutive patients who completed treatment with multibracket appliances were included in the study. All patients provided written consent, and no patients or cephalograms were excluded. The sample size was estimated as the average rotation error of the pilot experiment and analog method (19). The sample size of the Bland–Altman method comparison with a mean rotation error *θ* of 0.31°, an alpha error of 0.05, a beta error of 0.20, and a maximum allowable difference of 1° resulted in a minimum of 24 patients (27). Accuracy describes herein the comparison of mean value of a new method compared with another or the best method, whereas the precision describes the variation of the measurement around the mean value.

### Interventions

All patients were treated with self-ligating braces (SPEED system) in combination with additional appliances for orthodontic, craniofacial, and general dentistry-related problems. Cephalograms before (T1) and at the end of the active treatment (T2) were collected (S.S.) and used for superimposition. Parameters of 62 kV, 16 mA, and 0.3 s were used for image acquisition by a ProMax 2D Digital (Planmeca, Helsinki, Finland) one-shot cephalostat with a sensor having a pixel size of 139 μm. The images were exported as lossless JPEG and calibrated on the true size of the mid-sagittal plane by the magnification factor of 1.13. The landmarks were placed in a coordinate system (x,y): The sella (s; the center of the sella turcica), articulare (ar), Walker's Point (wp; intersection of the anterior wall of the sella turcica and the lower midcontour of both processi clinoidei), the supraorbitale (sor; intersection of the inner contour of the anterior cranial fossa and the middle-contour of both orbital roofs), the nasion (n; the most anterior limit of the frontonasal suture), the spina nasalis anterior (spa), the pogonion (pg; the most anterior point on the bony chin), and the articulare (ar; intersection of the posterior margin of the ascending ramus and the outer margin of the cranial base). Ethical approval was granted by the Swiss Ethics Committee on Research Involving Humans 2023-01336.

### Methods of superimposition

**Figure 1:**
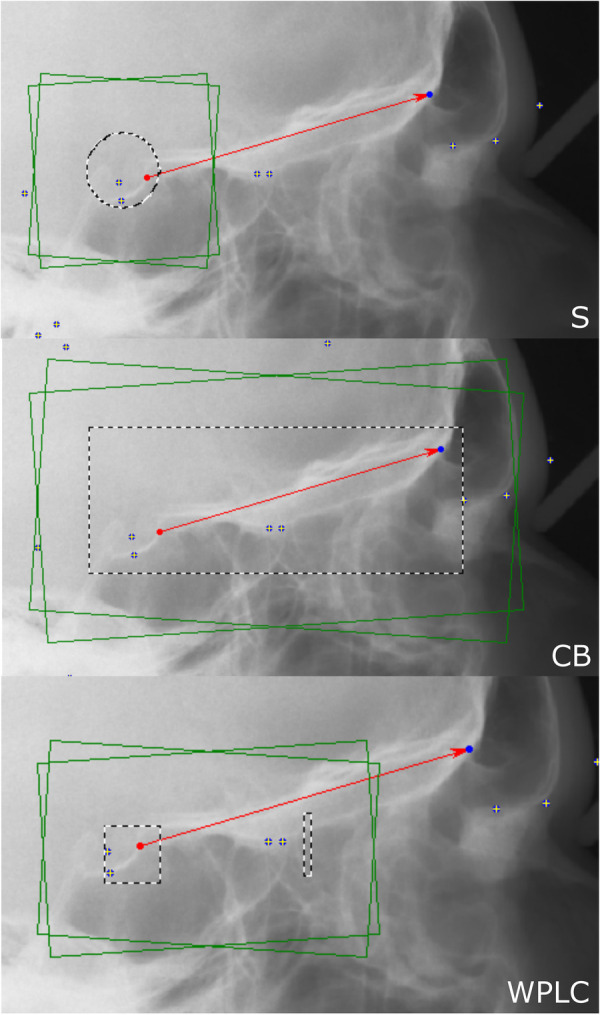
For digital image correlation using the sella method (**S**, left), a circle including the sella (**S**, dashed line) is used. For the cranial base method (**CB**, middle), a rectangle including the whole cranial base (dashed line) is used. For the Walkers’ point and lamina cribrosa method (WPLC, right), a square including the anterior wall of sella and the lower half of the processi clinoidei and a separate vertical rectangle (25% the size of the square) just anterior to the middle cranial fossa including the lamina cribrosa and the fronto-ethmoidal crests are used.

Cephalometric images were superimposed using DIC in OnyxCeph3™ (Image Instruments GmbH, Chemnitz, Germany) with pre-alignment at Walker's point (the intersection point of the averaged lower contours of the anterior clinoid processes and the anterior wall of the sella) in the direction of the supraorbital region (the intersection of the cerebral contours of the anterior cranial fossa and the middle contour of both orbital roofs). A search range of 10 mm, angular range of 4°, and steps of 0.1° were used. The three methods differed according to the area used for superimposition (Figure 1). The first method (S) included a circular area including all contours of the sella, while the second method (CB) involved the entire cranial base; in the third method (WPLC), a square area including the anterior wall of the sella and the lower half of the processi clinoidei was combined with an area one-quarter the width of the square, including the frontoethmoidal crests and the cribriform plate. The reason for avoiding the contours of the median cranial fossa and processus zygomaticus ossis frontalis is that these structures change during growth up to the age of 12–14 years. Correlation coefficients were calculated for each horizontal and vertical rotational increment. The image correlation algorithm was run for each rotation step and returned the highest correlation.

### Measurements

Two observers (P.R. and J.D.) superimposed the cephalometric radiographs using all three methods and repeated them the following day using OnyxCeph3TM (version 3.2.51; Image Instruments, Chemnitz, Germany).

The linear equation, the slope calculated from two points, an angular value of the slope and the angular error were used: $\begin{array}{c} \;f(x)=m*x+a\\ m_{1}=(wp_{Y1}-\mathrm{so}b_{Y1})/(wp_{X1}-\mathrm{so}b_{X1})\\ \theta_{1}=\arctan(m_{1})\\ \;\theta_{T1-T2}=\arctan(m_{1})-\arctan(m_{2}) \end{array}$

The amount of rotation of the cephalometric image (*θ*) and the difference between repeated superimpositions (*θ*_T1–T2_) were calculated using the line between Walker's point (wp) and supraorbitale (sob). A linear function is solved to calculate the slope m1 of the line through wp and sob of the first superimposition. The slope m can be converted into an angular value *θ* using the inverse trigonometric function arctan. The angular error *θ*_T1–T2_ is calculated by subtracting the angle of the first superimposition from the second superimposition. Item 29 caused an error during digital image correlation which was eliminated by manual rotation of the image before correlation.

X- and y-coordinates of the cephalometric landmarks from the superimposed images were collected in the coordinate system of the reference image.

### Data analysis

Image rotation *θ* and cephalometric landmarks were analyzed using a multivariate repeated measurements model with fixed effects id, method, observer, and time. Variances were estimated using a random coefficient model including the interactions between patient and time, patient and observer, and patient and method, as well as a numerical indicator variable for each method using STATA 18.0 (StatCorp, College Station, Texas, USA) (28). Predictive margins and residuals were calculated and plotted. Inter-rater and intra-rater agreement were calculated with and without the exclusion of outlier item 29 using Prism 9 (GraphPad Software, Boston, MA, USA) and displayed as Bland–Altman plots (29, 30). Pearson correlation coefficients were calculated to test the strength of the relationship between differences in *θ*_T1–T2_ and all landmarks _T1–T2_.

## Results

The study population consisted of 12 females and 18 males with an average age of 15.3 (±1.64) at T2. The period between T1 and T2 was 2.1 ± 0.78 years. *P*-values less than 0.05 were considered significant.

### Image rotation during superimposition

When comparing the three methods using the multivariate model including random coefficients, there were no significant mean differences found between observers (*p* = 0.47), timepoints (*p *= 0.62), or interactions between method/observer/time (*p *= 0.72–0.97), method/observer (*p* = 0.18–0.85), method/time (*p *= 0.11–0.65), or observer/time (*p* = 0.15). The superimposed cephalogram was rotated significantly less in WPLC when compared with CB by 0.31° (CI: −0.56 to −0.07, *p* = 0.01), whereas the means of S vs. CB (*p *= 0.93) and S vs. WPLC (*p* = 0.13) did not differ. The precision of image rotation was *σ2 = *2.97 (±1.72°) using S, *σ*2 *= *0.37 (±0.61°) using SB, and *σ*2 *= *0.25 (±0.50°) using WPLC.

**Figure 2:**
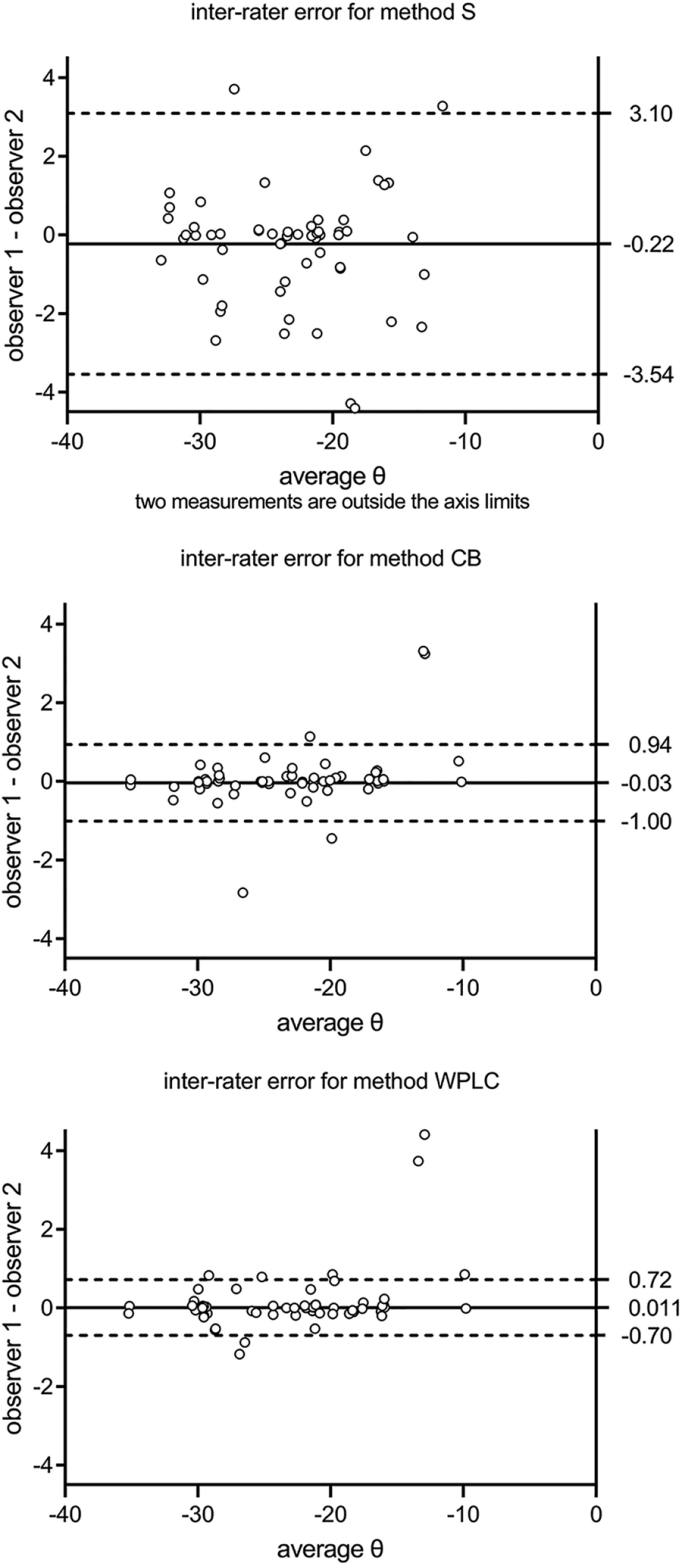
Inter-rater agreement is calculated as mean and 95% limits of agreement between two observers. S, sella; CB, cranial base; WPLC, Walker's point and lamina cribrosa.

Calculation of inter-rater agreement revealed no significant bias between observers for all three methods (*p* = 0.17–0.81 with and without exclusion of outlier item 29). The variance for item 29 was 14.39°, which was exceptionally high compared with all other items. The limits of agreement were from −4.92° to 5.10° for S, from −1.44° to 1.59° for CB, and from −1.46° to 1.76° for WPLC. When outlier item 29 was removed, the limits of agreement ranged from −3.54° to 3.10° for S, from −1.00° to 0.94° for CB, and from −0.70° to 0.72° for WPLC (Figure 2).

**Figure 3:**
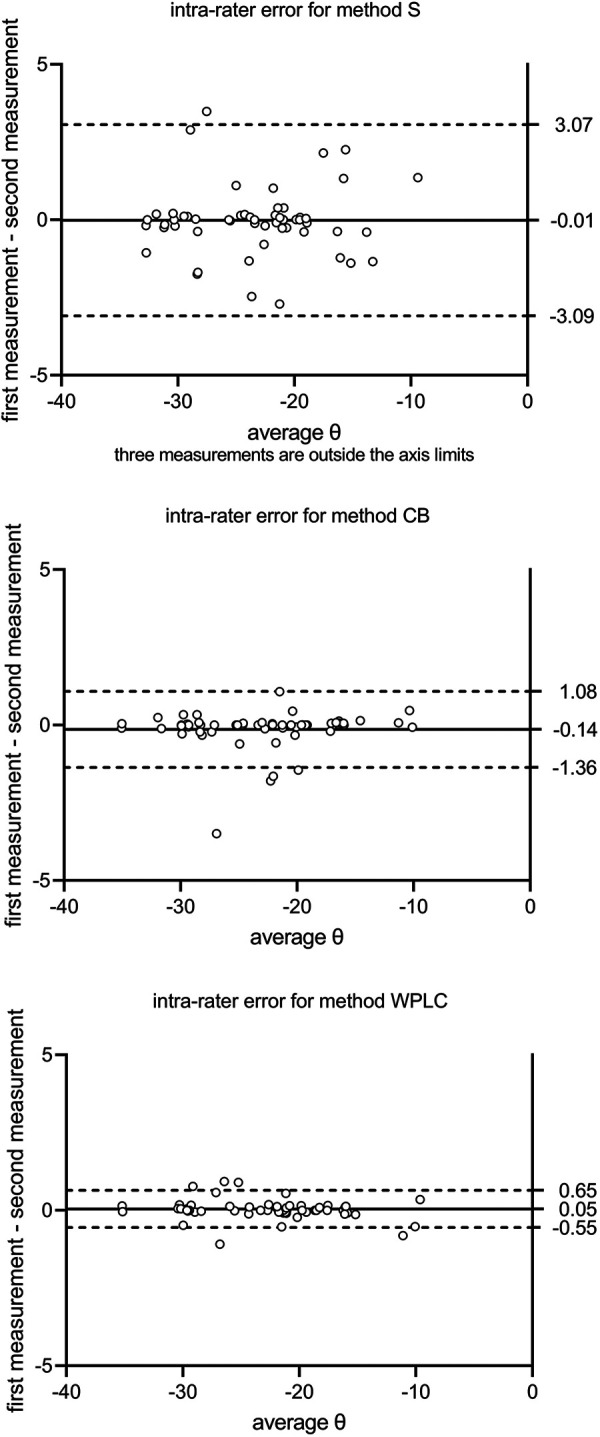
Intra-rater agreement is calculated as mean and 95% limits of agreement between the first and second measurements (T1 and T2). S, sella; CB, cranial base; WPLC, Walker's point and lamina cribrosa.

No significant bias was found for intra-rater agreement (difference in image rotation *θ*_T1–T2_ over *θ*_average_) between repeated measurements for all three methods (*p* = 0.10–0.96 with and without exclusion of outlier item 29, respectively). The limits of agreement were from −4.94° to 5.10° for S, from −1.44° to 0.77° for CB, and from −1.46° to 0.82° for WPLC. With the exclusion of outlier item 29, the limits of agreement were from −3.10° to 3.07° for S, from −1.36° to 1.08° for CB, and from −0.55° to 0.65° for WPLC (Figure 3).

The difference in image rotation *θ*_T1–T2_ was strongly correlated with some horizontal error of the landmark error x_T1–T2_ as well as the vertical error of most landmark errors y_T1–T2_ for all three methods (Table 1).

**Table 1:** Correlation between the image rotation error and the error of the landmarks.

Landmark	Method	*n*	Pearson *r*	*r* ^2^	Strength of relationship	Strength of relationship	*r* ^2^	Pearson *r*	*n*	Method	Landmark
x Walker's point	S	60	−0.50	0.25	weak	strong	0.87	0.93	60	S	y Walker's point
	CB	60	−0.38	0.15	no	strong	0.81	0.90	60	CB	
	WPLC	60	0.21	0.04	no	strong	0.78	0.88	60	WPLC	
x sella	S	60	−0.67	0.45	weak	no	0.36	−0.60	60	S	y sella
	CB	60	−0.46	0.21	no	strong	0.80	−0.90	60	CB	
	WPLC	60	0.13	0.02	no	weak	0.30	−0.55	60	WPLC	
x nasion	S	60	0.50	0.25	weak	strong	0.81	0.90	60	S	y nasion
	CB	60	0.34	0.12	no	strong	0.78	0.88	60	CB	
	WPLC	60	0.43	0.19	no	strong	0.75	0.86	60	WPLC	
x anterior nasal spine	S	60	−0.98	0.95	strong	strong	0.87	0.94	60	S	y anterior nasal spine
	CB	60	−0.94	0.89	strong	strong	0.83	0.91	60	CB	
	WPLC	60	−0.67	0.45	weak	strong	0.81	0.90	60	WPLC	
x articulare	S	60	−0.98	0.96	almost Perfect	strong	0.63	−0.79	60	S	y articulare
	CB	60	−0.90	0.82	strong	strong	0.87	−0.94	60	CB	
	WPLC	60	−0.61	0.37	weak	strong	0.56	−0.75	60	WPLC	
x pogonion	S	60	−1.00	0.99	almost perfect	moderate	0.87	0.93	60	S	y pogonion
	CB	60	−0.98	0.97	almost perfect	strong	0.81	0.90	60	CB	
	WPLC	60	−0.92	0.84	strong	moderate	0.78	0.88	60	WPLC	

The difference in image rotation between the first and second measurements (*θ*_T1–T2_) is highly- to perfectly-correlated with the horizontal component (x_T1–T2_) of the anterior nasal spine, articulare, and pogonion landmarks. Almost all y_T1–T2_ values of vertical landmarks are strongly correlated with the difference in image rotation (*θ*_T1–T2_) between the first and second measurements. S, sella; CB, cranial base; WPLC, Walker's point and lamina cribrosa.

### Accuracy and precision of cephalometric landmarks

The accuracy of landmarks differed between methods between −0.56 and 0.38 mm horizontally and between −0.23 and 0.040 mm vertically. Precision ranged from ±0.21 to 2.99 mm horizontally and from ±0.19 to 2.08 mm vertically (Table 2). The methods explained 59.6%–99.5%, repeated measurements explained 0.0%–2.2%, and observers explained 0.0%–36.5% of the variance, leaving a residual variance of 0.0%–17.4%.

**Table 2:** Accuracy and precision of the landmarks for each method.

	Method	Accuracy					Precision				
								Contribution of random coefficients to total variance			
		Mean	Comparison	*p*	Lower 95% CI	Upper 95% CI		Method	Time	Observer	Residual
x Walker's point	S	0.25 mm	vs. CB	<0.01	0.16 mm	0.33 mm	±0.22 mm	90.3% (±0.20 mm)	0.0% (±0.00 mm)	3.1% (±0.01 mm)	6.7% (±0.01 mm)
	CB	−0.15 mm	vs. WPLC	0	−0.23 mm	−0.06 mm	±0.23 mm	91.5% (±0.21 mm)	0.0% (±0.00 mm)	2.7% (±0.01 mm)	5.8% (±0.01 mm)
	WPLC	−0.10 mm	vs. S	0.02	−0.02 mm	−0.18 mm	±0.22 mm	90.4% (±0.20 mm)	0.0% (±0.00 mm)	3.0% (±0.01 mm)	6.5% (±0.01 mm)
y Walker's point	S	−0.22 mm	vs. CB	0.01	−0.38 mm	−0.06 mm	±0.77 mm	96.2% (±0.74 mm)	0.0% (±0.00 mm)	3.1% (±0.02 mm)	0.7% (±0.01 mm)
	CB	0.19 mm	vs. WPLC	<0.01	0.09 mm	0.28 mm	±0.32 mm	78.3% (±0.25 mm)	0.0% (±0.00 mm)	17.9% (±0.06 mm)	3.8% (±0.01 mm)
	WPLC	0.03 mm	vs. S	0.7	−0.13 mm	0.19 mm	±0.24 mm	59.9% (±0.14 mm)	0.0% (±0.00 mm)	33.0% (±0.08 mm)	7.0% (±0.02 mm)
x sella	S	0.25 mm	vs. CB	<0.01	0.17 mm	0.33 mm	±0.22 mm	96.8% (±0.22 mm)	0.0% (±0.00 mm)	0.0% (±0.00 mm)	3.2% (±0.01 mm)
	CB	−0.14 mm	vs. WPLC	<0.01	−0.22 mm	−0.05 mm	±0.23 mm	97.0% (±0.23 mm)	0.0% (±0.00 mm)	0.0% (±0.00 mm)	3.0% (±0.01 mm)
	WPLC	−0.11 mm	vs. S	<0.01	−0.19 mm	−0.03 mm	±0.21 mm	96.4% (±0.20 mm)	0.0% (±0.00 mm)	0.0% (±0.00 mm)	3.6% (±0.01 mm)
y sella	S	−0.22 mm	vs. CB	0.01	−0.40 mm	−0.05 mm	±0.82 mm	95.9% (±0.79 mm)	0.0% (±0.00 mm)	3.5% (±0.03 mm)	0.5% (±0.00 mm)
	CB	0.21 mm	vs. WPLC	<0.01	0.11 mm	0.32 mm	±0.36 mm	78.6% (±0.28 mm)	0.0% (±0.00 mm)	18.6% (±0.07 mm)	2.8% (±0.01 mm)
	WPLC	0.01 mm	vs. S	0.94	−0.16 mm	0.18 mm	±0.26 mm	59.6% (±0.16 mm)	0.0% (±0.00 mm)	35.1% (±0.09 mm)	5.3% (±0.01 mm)
x nasion	S	0.25 mm	vs. CB	<0.01	0.11 mm	0.39 mm	±0.40 mm	89.7% (±0.36 mm)	1.4% (±0.01 mm)	7.7% (±0.03 mm)	1.2% (±0.00 mm)
	CB	−0.21 mm	vs. WPLC	<0.01	−0.35 mm	–0.08 mm	±0.31 mm	83.0% (±0.26 mm)	2.3% (±0.01 mm)	12.6% (±0.04 mm)	2.0% (±0.01 mm)
	WPLC	−0.04 mm	vs. S	0.59	−0.18 mm	0.10 mm	±0.32 mm	83.9% (±0.27 mm)	2.2% (±0.01 mm)	11.9% (±0.04 mm)	1.9% (±0.01 mm)
y nasion	S	−0.20 mm	vs. CB	0.26	−0.55 mm	0.15 mm	±1.78 mm	98.9% (±1.76 mm)	0.0% (±0.00 mm)	0.3% (±0.01 mm)	0.8% (±0.01 mm)
	CB	−0.14 mm	vs. WPLC	0.1	−0.31 mm	0.03 mm	±0.40 mm	77.5% (±0.31 mm)	0.0% (±0.00 mm)	5.7% (±0.02 mm)	16.8% (±0.07 mm)
	WPLC	0.34 mm	vs. S	0.06	−0.01 mm	0.69 mm	±0.43 mm	81.0% (±0.35 mm)	0.0% (±0.00 mm)	4.8% (±0.02 mm)	14.2% (±0.06 mm)
x anterior nasal spine	S	0.21 mm	vs. CB	0.11	−0.05 mm	0.48 mm	±1.37 mm	99.2% (±1.36 mm)	0.0% (±0.00 mm)	0.1% (±0.00 mm)	0.7% (±0.01 mm)
	CB	0.08 mm	vs. WPLC	0.14	−0.02 mm	0.18 mm	±0.36 mm	89.0% (±0.32 mm)	0.0% (±0.00 mm)	1.4% (±0.01 mm)	9.5% (±0.03 mm)
	WPLC	−0.29 mm	vs. S	0.03	−.55 mm	−0.03 mm	±0.27 mm	79.9% (±0.21 mm)	0.0% (±0.00 mm)	2.6% (±0.01 mm)	17.4% (±0.05 mm)
y anterior nasal spine	S	−0.21 mm	vs. CB	0.33	−0.62 mm	0.21 mm	±2.08 mm	98.9% (±2.06 mm)	0.0% (±0.00 mm)	0.4% (±0.01 mm)	0.7% (±0.01 mm)
	CB	−0.20 mm	vs. WPLC	0.05	−0.39 mm	0.00 mm	±0.48 mm	80.2% (±0.39 mm)	0.0% (±0.00 mm)	7.4% (±0.04 mm)	12.4% (±0.06 mm)
	WPLC	0.40 mm	vs. S	0.06	−0.01 mm	0.82 mm	±0.50 mm	81.9% (±0.41 mm)	0.0% (±0.00 mm)	6.8% (±0.03 mm)	11.3% (±0.06 mm)
x pogonion	S	0.19 mm	vs. CB	0.54	−0.41 mm	0.78 mm	±2.99 mm	98.5% (±2.95 mm)	0.0% (±0.00 mm)	1.0% (±0.03 mm)	0.5% (±0.02 mm)
	CB	0.38 mm	vs. WPLC	0.01	−0.03 mm	1.15 mm	±0.91 mm	83.9% (±0.76 mm)	0.0% (±0.00 mm)	10.6% (±0.10 mm)	5.6% (±0.05 mm)
	WPLC	−0.56 mm	vs. S	0.06	−1.15 mm	0.03 mm	±0.71 mm	73.1% (±0.52 mm)	0.0% (±0.00 mm)	17.6% (±0.12 mm)	9.3% (±0.07 mm)
y pogonion	S	−0.23 mm	vs. CB	0.23	−0.60 mm	0.14 mm	±1.88 mm	99.3% (±1.87 mm)	0.0% (±0.00 mm)	0.3% (±0.01 mm)	0.4% (±0.01 mm)
	CB	−0.13 mm	vs. WPLC	0.15	−0.31 mm	0.05 mm	±0.44 mm	87.1% (±0.38 mm)	0.0% (±0.00 mm)	5.8% (±0.03 mm)	7.0% (±0.03 mm)
	WPLC	0.36 mm	vs. S	0.06	−0.01 mm	0.73 mm	±0.45 mm	88.1% (±0.40 mm)	0.0% (±0.00 mm)	5.4% (±0.02 mm)	6.5% (±0.03 mm)
x articulare	S	0.21 mm	vs. CB	0.08	−0.03 mm	0.45 mm	±1.23 mm	99.5% (±1.22 mm)	0.0% (±0.00 mm)	0.0% (±0.00 mm)	0.5% (±0.01 mm)
	CB	0.05 mm	vs. WPLC	0.29	−0.04 mm	0.15 mm	±0.34 mm	93.4% (±0.32 mm)	0.0% (±0.00 mm)	0.0% (±0.00 mm)	6.6% (±0.02 mm)
	WPLC	−0.26 mm	vs. S	0.03	−0.49 mm	−0.03 mm	±0.24 mm	86.1% (±0.20 mm)	0.0% (±0.00 mm)	0.0% (±0.00 mm)	13.9% (±0.03 mm)
y articulare	S	−0.23 mm	vs. CB	0.05	−0.46 mm	0.00 mm	±1.08 mm	94.9% (±1.03 mm)	0.0% (±0.00 mm)	3.8% (±0.04 mm)	1.3% (±0.01 mm)
	CB	0.29 mm	vs. WPLC	<0.01	0.15 mm	0.43 mm	±0.48 mm	73.7% (±0.35 mm)	0.0% (±0.00 mm)	19.5% (±0.09 mm)	6.8% (±0.03 mm)
	WPLC	−0.06 mm	vs. S	0.62	−0.28 mm	0.17 mm	±0.35 mm	50.9% (±0.18 mm)	0.0% (±0.00 mm)	36.5% (±0.13 mm)	12.6% (±0.04 mm)

Reproducibility was assessed as accuracy between methods and precision for each method was assessed with a multivariate model including random coefficients. Abbreviations: S, sella; CB, cranial base; WPLC, Walker’s point and lamina cribrosa.

**Figure 4:**
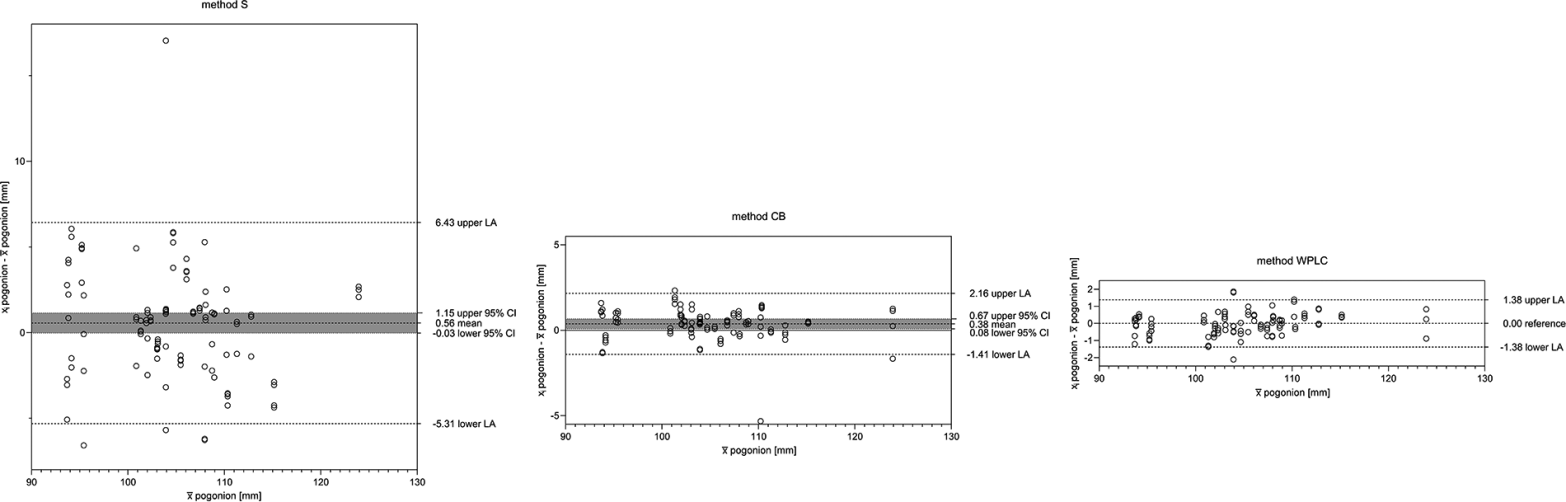
The difference plot for more than two groups. The mean (x¯) is plotted against the difference to the mean (xi-x¯) using a random coefficient model. The WPLC method with the lowest variance is used as a reference. S, sella; CB, cranial base; WPLC, Walker's point and lamina cribrosa.

The precision of S ranged from ±0.22 to 2.99 mm horizontally in the order wp < s < n < ar < ans < pg, and from ±0.77 to 2.08 mm vertically in the order wp < s < ar < n < pg < ans. When comparing S to CB, significant shifts were observed in landmarks x_wp, y_wp, x_s, y_s, x_n, and y_ar. A comparison of S to WPLC revealed a significant horizontal shift for x_wp, x_s, x_ans, and x_ar, but no significant vertical shifts. The precision of CB ranged horizontally from ±0.23 to 0.91 mm in the order x_wp < x_s < x_ar < x_ans < x_pg, and vertically from ±0.32 to 0.44 mm in the order y_wp < y_ar < y_s < y_n < y_ans < y_pg. A significant shift between CB and WPLC was observed for landmarks x_wp, y_wp, x_s, y_s, x_n, y_ans, x_pg, and y_ar. The precision of WPLC ranged horizontally from ±0.21 to 0.71 mm in the order s < wp < ar < ans < n < pg and vertically from ±0.24 to 0.50 mm in the order wp < s < ar < n < pg < ans (Table 2 and Figure 4).

**Figure 5:**
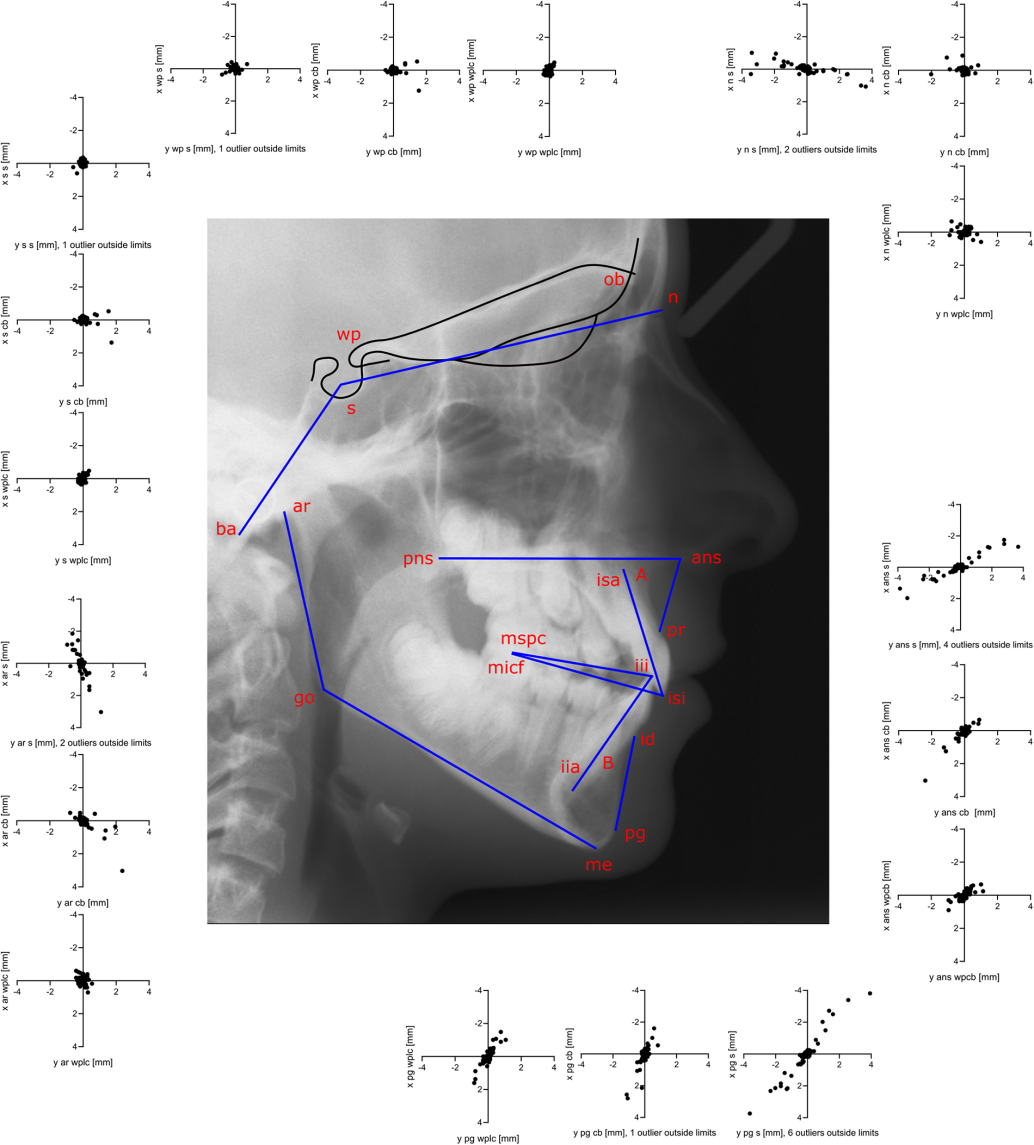
The second measurement is subtracted from the first measurement (T1–T2) to display the precision of every landmark and the correlations of the horizontal x_T1–T2_ and vertical y_T1–T2_ components. Precision is best for landmarks near the cranial base. Correlation between horizontal and vertical error for nasion, anterior nasal spine, pogonion, and articulare may be an effect of rotational error during superimposition and this increases with the distance between the landmark and the center of rotation.

The differences between the first and second measurements were correlated with the horizontal x_T1–T2_ and vertical y_T1–T2_ components for landmarks distant from the structures used for superimposition (Table 3). Each landmark with a strong correlation was also correlated with the difference in image rotation *θ*_T1–T2_ (Figure 5 and Table 1).

**Table 3:** Correlation between horizontal and vertical error of the landmarks.

			Horizontal																	
			Method S						Method CB						Method WPLC					
		r2	x wp_T1–T2_	x s_T1–T2_	x n_T1–T2_	x ans_T1–T2_	x pog_T1–T2_	x ar_T1–T2_	x wp_T1–T2_	x s_T1–T2_	x n_T1–T2_	x ans_T1–T2_	x pog_T1–T2_	x ar_T1–T2_	x wp_T1–T2_	x s_T1–T2_	x n_T1–T2_	x ans_T1–T2_	x pog_T1–T2_	x ar_T1–T2_
Vertical	Method S	y wp_T1–T2_	0.07	0.19	0.48	0.73	0.82	0.73	0.01	0.02	0.00	0.03	0.03	0.03	0.00	0.00	0.00	0.02	0.02	0.01
		y s_T1–T2_	0.56	0.68	0.02	0.50	0.42	0.53	0.00	0.00	0.01	0.00	0.00	0.00	0.00	0.00	0.00	0.00	0.00	0.00
		y n_T1–T2_	0.05	0.14	0.47	0.66	0.76	0.65	0.01	0.01	0.00	0.02	0.03	0.02	0.00	0.00	0.01	0.02	0.03	0.01
		y ans_T1–T2_	0.07	0.19	0.45	0.74	0.83	0.73	0.00	0.01	0.00	0.02	0.02	0.02	0.00	0.00	0.00	0.02	0.02	0.01
		y pog_T1–T2_	0.07	0.19	0.48	0.73	0.82	0.73	0.01	0.02	0.00	0.03	0.03	0.03	0.00	0.00	0.00	0.02	0.02	0.01
		y ar_T1–T2_	0.56	0.74	0.00	0.76	0.69	0.78	0.00	0.00	0.01	0.00	0.00	0.00	0.00	0.00	0.00	0.00	0.00	0.00
	Method CB	y wp_T1–T2_	0.00	0.01	0.03	0.01	0.01	0.01	0.30	0.38	0.01	0.85	0.85	0.83	0.02	0.02	0.02	0.00	0.01	0.00
		y s_T1–T2_	0.02	0.00	0.10	0.01	0.01	0.01	0.03	0.07	0.21	0.63	0.73	0.55	0.02	0.01	0.02	0.01	0.00	0.01
		y n_T1–T2_	0.00	0.01	0.02	0.01	0.01	0.01	0.26	0.34	0.01	0.82	0.82	0.79	0.02	0.03	0.02	0.00	0.02	0.00
		y ans_T1–T2_	0.00	0.01	0.02	0.01	0.01	0.01	0.24	0.32	0.03	0.84	0.87	0.80	0.01	0.02	0.01	0.00	0.01	0.00
		y pog_T1–T2_	0.00	0.01	0.03	0.01	0.01	0.01	0.30	0.38	0.01	0.85	0.85	0.83	0.02	0.02	0.02	0.00	0.01	0.00
		y ar_T1–T2_	0.01	0.00	0.09	0.01	0.02	0.01	0.04	0.08	0.22	0.70	0.81	0.60	0.01	0.01	0.02	0.01	0.00	0.01
	Method WPLC	y wp_T1–T2_	0.00	0.00	0.00	0.01	0.01	0.01	0.00	0.00	0.01	0.01	0.01	0.01	0.00	0.00	0.06	0.56	0.80	0.48
		y s_T1–T2_	0.03	0.02	0.00	0.00	0.00	0.00	0.00	0.00	0.00	0.00	0.01	0.00	0.23	0.18	0.33	0.00	0.12	0.00
		y n_T1–T2_	0.00	0.00	0.01	0.02	0.02	0.02	0.00	0.00	0.01	0.00	0.01	0.00	0.00	0.01	0.05	0.57	0.79	0.50
		y ans_T1–T2_	0.00	0.00	0.01	0.01	0.02	0.01	0.00	0.00	0.01	0.01	0.01	0.01	0.00	0.00	0.06	0.59	0.84	0.51
		y pog_T1–T2_	0.00	0.00	0.00	0.01	0.01	0.01	0.00	0.00	0.01	0.01	0.01	0.01	0.00	0.00	0.06	0.56	0.80	0.48
		y ar_T1–T2_	0.02	0.01	0.00	0.00	0.00	0.00	0.00	0.00	0.00	0.00	0.00	0.00	0.18	0.13	0.33	0.08	0.33	0.06

*r*^2^ values larger than 0.5 are highlighted in blue. A correlation between x_T1–T2_ and y_T1–T2_ components indicates an influence of error in image rotation on landmark precision. A value of 0.8 indicates that 55% of the standard deviation is potentially explained by an image rotation error. Landmarks distant from the center of rotation (n, ans, pog, and ar) are more affected by rotational error than wp or s. Methods S and CB are more affected by rotational error than WPLC. S, sella; CB, cranial base; WPLC, Walker’s point and lamina cribrosa.

## Discussion

The analysis of dentofacial growth as well as orthodontic or surgical treatment in longitudinal studies depends on precise and accurate measurement methods and the presence of stable structures. Progress in computed cephalometric imaging and DIC has enabled the digital implementation of “The Structural Method” (8). Cephalometric structural DIC enables treatment and growth analysis not only for research and case studies but also for clinical applications when longitudinal data are available. Cephalometric DIC was tested in this study at three different areas of the cranial base.

DIC with WPLC uses the most stable structures of the cranial base; it had ±0.64° for image rotation *θ* and the best intra-rater and inter-rater agreement, making it the most precise method. When using CB with the entire cranial base, the precision of *θ* decreased to ±0.74°, which is close to the ±0.71° found for manual superimpositions (19) and is within an acceptable range. We do not recommend using DIC with the entire sella, as the precision of S was unreliable (± 1.77°). The most important source of variance was the method, followed by the observer, whereas residual error and reproducibility were negligible. One advantage of DIC is that it is not dependent on landmarks, as these were only used to pre-align the images. The WPLC method had the highest precision and accuracy, making it suitable for analyzing growth changes and treatment effects of maxillofacial orthodontic or surgical treatments. The lack of accurate methods for facial growth analysis and low quality of research was described (26). The low quality of most studies may contribute to the lack of significant differences in accuracy between the methods analyzed. Our study showed that DIC with the WPLC method is accurate and the precision is better than what has been described in the literature (26). Superimpositions with DIC on growth-stable structures are accurate with high precision and, when using the WPLC method, slightly better than the traditional method.

The central midsagittal part of the cranial base was considered to be stable in the adolescents examined because the frontosphenoidal, sphenoethmoidal, and sphenotemporal sutures were already closed at T1 (14). The anterior part of the internal surface of the sella turcica, frontoethmoidal crests, and cribriform plate become stable very early in life (8, 10, 11). Adjacent structures such as the clivus (point basion) with synchondrosis sphenooccipitalis, the posterior part of the sella turcica (point sella), and the frontal sinus (point nasion) undergo significant changes up to adulthood (8, 11, 13, 31). Growth-related changes in unstable structures impair the accuracy of superimposition (32), which may explain the significant bias between CB and WPLC. A rotation of 0.31° and a 0.19-mm shift down at Walker's point was observed with CB when compared with WPLC, which does not include parts of the orbit for superimposition (8, 33, 34); this indicates that apposition at the orbital floor and development of the frontal sinus occurs. Development of the frontal sinus possibly caused a 0.15-mm posterior shift in CB when compared with WPLC; similar patterns were observed at the sellar and nasion points. Further, it is preferable to use central midsagittal structures for DIC and exclude adjacent structures that are subject to growth-related changes, which could reduce precision and cause bias. The inclusion of paramedian structures (planum sphenoidale, anterior and middle cranial fossa, and orbits) in CB could reduce precision, as double contours are affected by magnification and projection errors during cephalometry.

The DIC algorithm failed only for item 29, likely because of the large discrepancy between the head positions at T1 and T2. Manual adjustment of the T2 image resolved the error; however, the measurements for this item were outliers. Recalculating images before DIC may reduce its reliability and should be avoided. One limitation of DIC are incorrect superimpositions, which occur with poor radiographic image quality or anatomical anomalies. A visual check by a trained professional is therefore required to validate the result. For outliers such as item 29, there should be an option in the software to make the overlay manually with semi-transparent images.

**Figure 6:**
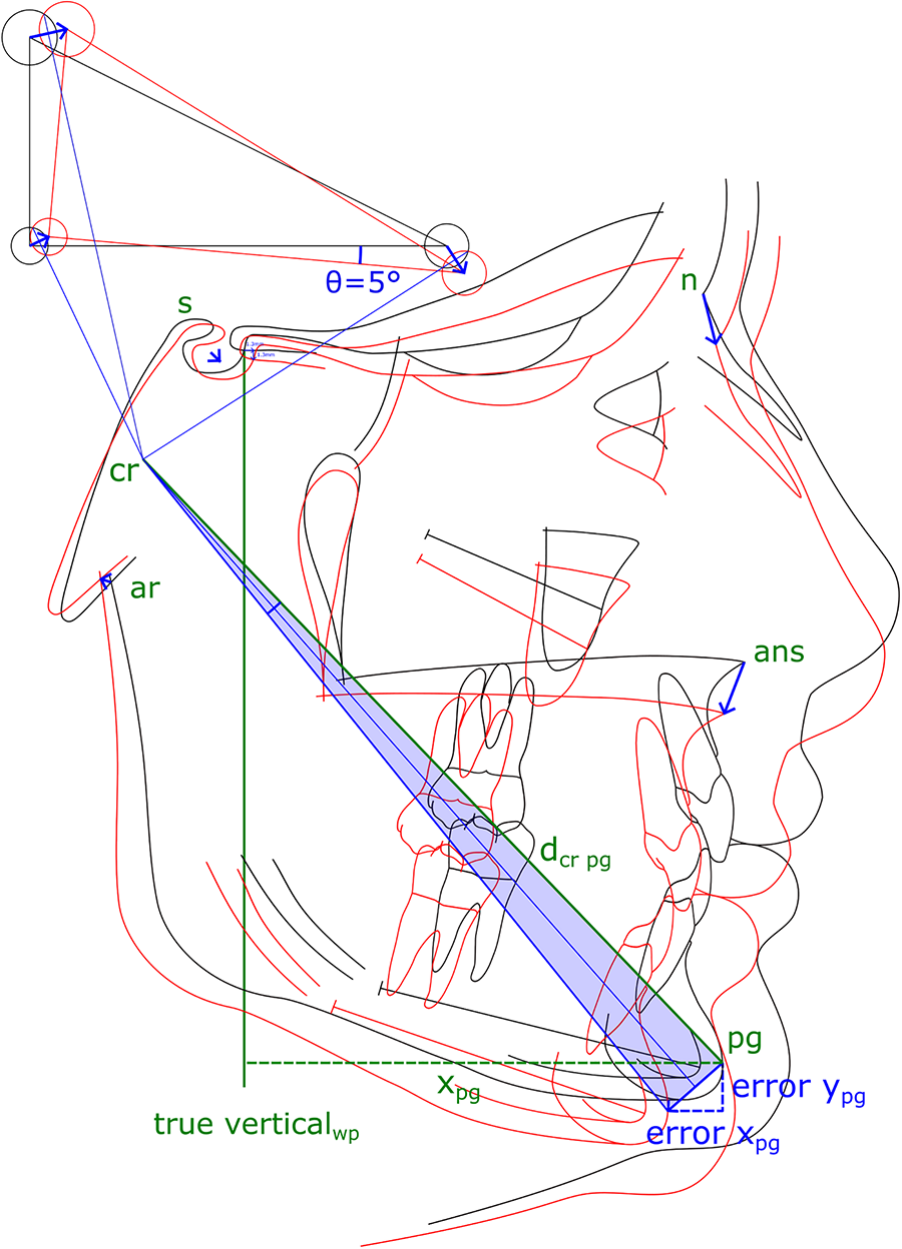
The second cephalogram is superimposed for a first time (black tracing) and then replicated the day after (red tracing). An example shows an assumed error of 5° of rotation around the Walker's point, shifted 1.3 mm right and 1.3 mm down. The location of the center of rotation (cr) is constructed and its location varies depending on rotation and shifts. Not all cephalometric points are equally affected by the superposition error (blue arrows). The resulting error in treatment and growth analysis increases with the distance from the center of rotation (dcr). The error increases with the distance of a landmark to the center of rotation; as an example, for pogonion by errorpg = 2*dcr pg*sin(*θ*Error/4).

**Figure 7:**
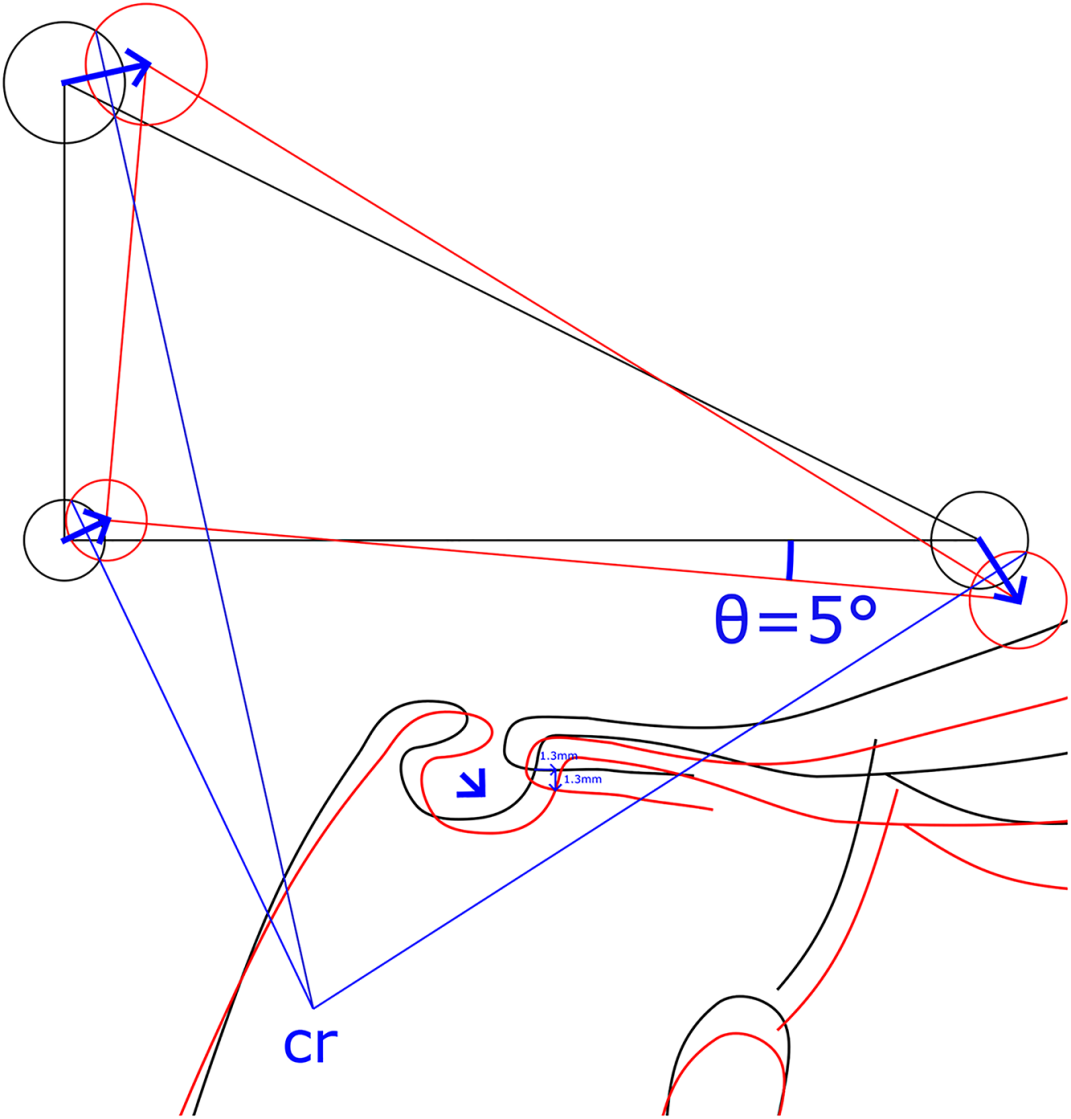
Construction of the center of rotation (cr). The rotation error of a superimposition *θ* remains constant independent of the location of the center of rotation and translational shift. The reproducibility of a general superimposition on the cranial base is best described as rotation *θ*Error and translation xError and yError at a chosen point (such as Walker's point). Depending on the magnitude and direction of the superimposition error, the location of the center of rotation changes.

Superimposition using S is not recommended, as precision decreased from ±0.22 mm at x_wp to ±2.99 mm at x_pg, indicating a strong rotational error. A decrease in precision from x_wp to x_pg was also found in CB from ±0.23 to 0.91 mm and from ±0.22 to 0.71 mm in WPLC. Point pg had the largest variance and the longest distance to the stable structures (Figure 6). An error in alignment and rotation during superimposition would likely result in an individual center of rotation near the stable structures at the cranial base (Figure 7).

Measuring the reproducibility of image rotation during DIC is a good quality control method because rotational error was highly correlated with a decrease in landmark precision. It is important in agreement studies comparing methods to investigate both accuracy (low bias, close to zero) and precision (low variance), as trueness depends on the measurement method used and is unknown. It should also be possible to apply DIC to 3D data with the advantage that no landmarks or surface-segmentations are required and image data is superimposed directly. However, this would involve standardized 3D images with high-resolution including growth-stable structures at the cranial base. For radiation protection, CBCTs are only indicated in exceptional cases when the treatment of severe asymmetries or craniofacial deformities is planned. Sagittal or vertical malocclusions are more common and the advantage for the patient from additional longitudinal 3D radiographs has not been proven.

Future research in 2D or 3D craniofacial growth analysis should focus on minimizing the rotation error during superimposition to further improve the precision of the analysis. It is difficult to propose detailed improvements as the exact algorithm used for DIC has not been disclosed. A potential future approach could be an iterative algorithm in which structures for horizontal (anterior wall of sella) and vertical displacements (lamina cribrosa and the fronto-ethmoidal crests) are weighted and maximized separately. An increase in the pixel resolution of the sensor or a longer film-focus distance could further improve precision of DIC.

In conclusion, superimposition of staged computed cephalometric radiographs using DIC with WPLC was the most precise method for growth and orthodontic or maxillofacial treatment analysis, demonstrating that it is a practical method for use in clinical applications. Including unstable anatomical structures in the superimposition, as in CB, caused bias and lowered precision to the level of manual superimposition. Using the entire contour of the sella turcica for DIC lowered the precision to below a clinically acceptable level. Rotational errors during DIC were strongly correlated with landmark errors. The precision of the cephalometric landmarks was highest near the cranial base and decreased with increasing distance from the center of rotation. Using a reference grid with true vertical and true horizontal lines from Walker's point after structural superimposition of serial cephalometric radiographs using WPLC was the most valid method for analyzing longitudinal dental and craniofacial changes. Further research to improve structural superposition should focus on reducing the rotational error of DIC.

## Data Availability

The raw data supporting the conclusions of this article will be made available by the authors, without undue reservation.
